# Role of NADPH Oxidase versus Neutrophil Proteases in Antimicrobial Host Defense

**DOI:** 10.1371/journal.pone.0028149

**Published:** 2011-12-07

**Authors:** R. Robert Vethanayagam, Nikolaos G. Almyroudis, Melissa J. Grimm, David C. Lewandowski, Christine T. N. Pham, Timothy S. Blackwell, Ruta Petraitiene, Vidmantas Petraitis, Thomas J. Walsh, Constantin F. Urban, Brahm H. Segal

**Affiliations:** 1 Department of Medicine, Roswell Park Cancer Institute, Buffalo, New York, United States of America; 2 Department of Medicine, School of Medicine and Biomedical Sciences, University at Buffalo, Buffalo, New York, United States of America; 3 Department of Medicine, Washington University School of Medicine, St Louis, Missouri, United States of America; 4 Department of Medicine, Vanderbilt University School of Medicine, Nashville, Tennessee, United States of America; 5 Transplantation-Oncology Infectious Diseases Program, Weill Cornell University Medical Center, New York, New York, United States of America; 6 Department of Molecular Biology, Umeå University, Umeå, Sweden; 7 Department of Immunology, Roswell Park Cancer Institute, Buffalo, New York, United States of America; Louisiana State University, United States of America

## Abstract

NADPH oxidase is a crucial enzyme in mediating antimicrobial host defense and in regulating inflammation. Patients with chronic granulomatous disease, an inherited disorder of NADPH oxidase in which phagocytes are defective in generation of reactive oxidant intermediates (ROIs), suffer from life-threatening bacterial and fungal infections. The mechanisms by which NADPH oxidase mediate host defense are unclear. In addition to ROI generation, neutrophil NADPH oxidase activation is linked to the release of sequestered proteases that are posited to be critical effectors of host defense. To definitively determine the contribution of NADPH oxidase versus neutrophil serine proteases, we evaluated susceptibility to fungal and bacterial infection in mice with engineered disruptions of these pathways. NADPH oxidase-deficient mice (p47*^phox−/−^*) were highly susceptible to pulmonary infection with *Aspergillus fumigatus*. In contrast, double knockout neutrophil elastase (NE)^−/−^×cathepsin G (CG)^−/−^ mice and lysosomal cysteine protease cathepsin C/dipeptidyl peptidase I (DPPI)-deficient mice that are defective in neutrophil serine protease activation demonstrated no impairment in antifungal host defense. In separate studies of systemic *Burkholderia cepacia* infection, uniform fatality occurred in p47*^phox−/−^* mice, whereas NE^−/−^×CG^−/−^ mice cleared infection. Together, these results show a critical role for NADPH oxidase in antimicrobial host defense against *A. fumigatus* and *B. cepacia*, whereas the proteases we evaluated were dispensable. Our results indicate that NADPH oxidase dependent pathways separate from neutrophil serine protease activation are required for host defense against specific pathogens.

## Introduction

Chronic granulomatous disease is an inherited disorder of the NADPH oxidase in which phagocytes are defective in generation of superoxide anion and downstream reactive oxidant intermediates (ROIs). As a result of this defect, CGD patients suffer from recurrent life-threatening bacterial and fungal infections [1]. Among CGD patients, the degree of impairment of NADPH oxidase in neutrophils correlates with clinical disease severity [2]. Although the critical role of the phagocyte NADPH oxidase has been established for decades [3], the precise mechanisms by which NADPH oxidase mediates antimicrobial host defense are not well understood.

One mechanism by which NADPH oxidase can kill or injure pathogens is through the direct toxic effects of ROIs. The rapid activation of NADPH oxidase constitutes an emergency response to invading pathogens, and occurs in response to several stimuli such as formylated peptides, opsonized particles, integrin-dependent adhesion [4], [5], and ligation of specific pathogen recognition receptors (e.g., dectin-1 [6]). Syk and class IA phosphoinositide 3-kinases regulate neutrophil NADPH oxidase activation by *Aspergillus fumigatus* hyphae [7]. Upon activation of the oxidase, the cytoplasmic subunits p47*^phox^*, p67*^phox^*, and p40*^phox^* and Rac translocate to the membrane-bound heterodimer cytochrome comprised of gp91*^phox^* and p22 *^phox^*. Molecular oxygen is converted to superoxide anion, which can spontaneously or enzymatically be converted to cytotoxic metabolites, including H_2_0_2_, hydroxyl anion, and peroxynitrite anion. In neutrophils, myeloperoxidase catalyzes the conversion of H_2_0_2_ to hypohalous acid, which is potently microbicidal.

In addition to the direct injurious effect of ROIs against pathogens, NADPH oxidase may mediate host defense by activation of pre-formed neutrophil serine proteases. In resting neutrophils, the flavocytochrome subunits gp91*^phox^* and p22*^phox^* are principally located within the membrane of the secondary granules [8], [9]. Primary (azurophilic) and secondary granules fuse with the phagocytic vacuole, where their constituents can co-mingle. Reeves et al. [10] proposed that NADPH oxidase-dependent killing activity of neutrophils is primarily mediated by activation of neutrophil granular proteases [10]. They found that neutrophil NADPH oxidase activation leads to accumulation of ROIs within phagocytic vacuoles that is coupled to K+ influx and alkalinization of vacuoles [10]. In their model, changes in ionic concentration within vacuoles lead to solubilization and activation of antimicrobial neutrophil serine proteases, which, at rest, are held in an inactivated state within primary granules of neutrophils [10].

Neutrophil elastase (NE)-deficient mice have been shown to have an increased susceptibility to Gram-negative bacterial sepsis [11]. One mechanism by which NE can mediate host defense is by cleavage of pathogen virulence factors [12], [13]. While cathepsin-G (CG)-deficient mice have no obvious host defense defect to bacterial pathogens [14], the double knockout NE^−/−^×CG^−/−^ mice have reported deficits in both antibacterial and antifungal host defense [10], [15], leading investigators to posit that activation of neutrophil serine proteases is the major mechanism by which NADPH oxidase mediates host defense.

Neutrophil serine proteases, NE, CG, and proteinase 3 are synthesized as zymogens and their activation requires N-terminal processing activity of the lysosomal cysteine protease cathepsin C/dipeptidyl peptidase I (DPPI) [16]. Papillon-Lefèvre syndrome, a rare autosomal recessive disease resulting from loss-of-function mutations in the *DPPI* gene locus, is characterized by palmoplantar hyperkeratosis, periodontitis leading to loss of teeth, and severe bacterial infections, including liver abscesses [16], [17]. A case of hepatic zygomycosis was reported in a patient with Papillon-Lefèvre syndrome [18], pointing to a role for DPPI in antifungal host defense.

Taken together, there is evidence in humans and mice that NADPH oxidase and neutrophil serine proteases contribute critically to host defense. To definitively determine the relative contribution of NADPH oxidase versus neutrophil serine proteases against specific pathogens, we evaluated susceptibility of mice with engineered disruptions of these pathways when challenged with a clinical isolate of *Aspergillus fumigatus* or *Burkholderia cepacia*, two major pathogens encountered in CGD patients [19], [20], [21], [22], [23]. We found that protease-deficient mouse models did not recapitulate the severe immune impairment in CGD mice, and, in fact, demonstrated no obvious susceptibility to the tested pathogens compared to wildtype (WT) mice. Thus, our results suggest that NADPH oxidase directly mediates host defense against specific pathogens through neutrophil serine protease-independent pathways.

## Results

### Neutrophil elastase^−/−^×cathepsin G^−/−^ mice have intact host defense in pulmonary aspergillosis, whereas NADPH oxidase-deficient p47*^phox−/−^* mice succumb to infection

Invasive aspergillosis is a major cause of morbidity and mortality in CGD patients [19], [20], [21], [22], [23], and NADPH oxidase-deficient mice are similarly highly susceptible to experimental aspergillosis [24], [25], [26], [27], [28]. Prior studies showed that NE^−/−^×CG^−/−^ mice also had increased susceptibility compared to WT mice in a model of systemic aspergillosis [15]. We therefore asked whether NADPH oxidase-mediated antifungal host defense principally occurs via neutrophil serine protease-dependent pathways.

To address this question, we evaluated experimental aspergillosis in 3 groups of mice: 1) WT (C57BL/6) mice; 2) p47*^phox−/−^* (CGD) mice; and 3) NE^−/−^×CG^−/−^ mice. Prior studies from our and other laboratories showed that WT C57BL/6 mice can clear an intratracheal inoculum of *A. fumigatus* >10^7^ conidia (spores) per mouse [25], [29]. In contrast, we previously found that the LD_50_ in unmanipulated p47*^phox−/−^* mice is <10^4^ conidia/mouse [25]. We therefore selected a high inoculum (1.25×10^7^ conidia/mouse) and a low inoculum (1.25×10^4^ conidia/mouse) to evaluate susceptibility to aspergillosis.

**Figure 1:**
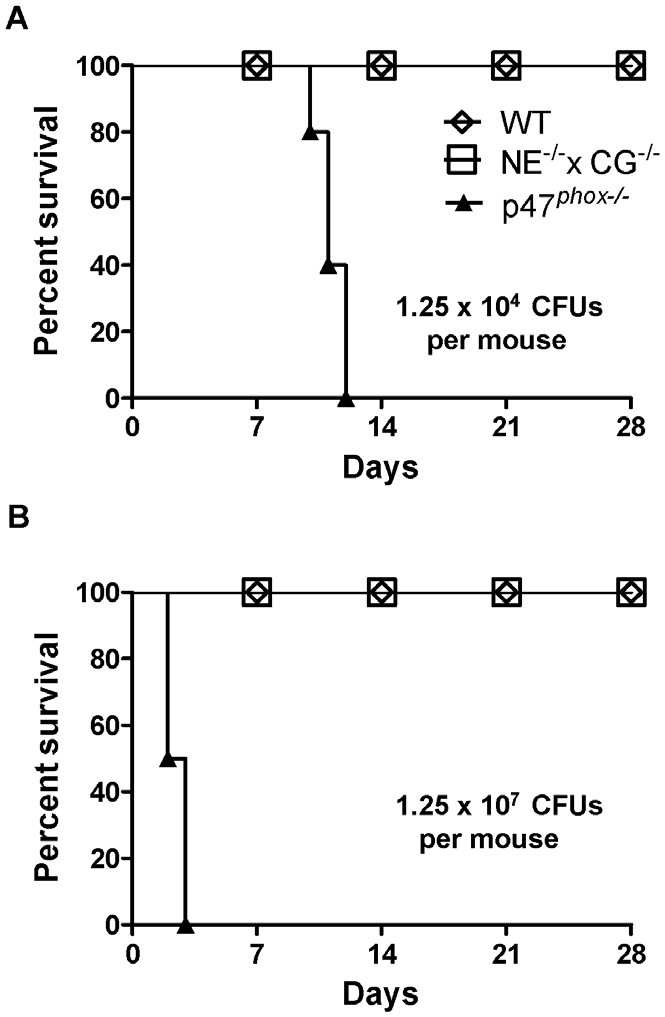
Kaplan-Meier survival curves of WT, p47*^phox^* ^**−/−**^ **, and NE^−/−^×CG^−/−^ mice after administration of** ***A. fumigatus*** **.** Mice were administered A) 1.25×10^4^ conidia or B) 1.25×10^7^ conidia by oropharyngeal aspiration. n = 5 mice per genotype per treatment. Log-rank analysis, p<0.0001 comparing WT with p47*^phox^* ^−/−^ mice and NE^−/−^×CG^−/−^ mice with p47*^phox^* ^−/−^ mice.

Following oropharyngeal challenge with 1.25×10^4^ conidia/mouse, all CGD mice died by 12 days, whereas WT and NE^−/−^×CG^−/−^ mice had uniform survival (Figure 1A). When administered the high inoculum (1.25×10^7^ conidia/mouse), all CGD mice died within 3 days, whereas all WT and NE^−/−^×CG^−/−^ mice survived (Figure 1B). Thus, the LD_50_ of the *A. fumigatus* inoculum is more than 1000-fold greater in NE^−/−^×CG^−/−^ mice versus CGD mice.

**Figure 2:**
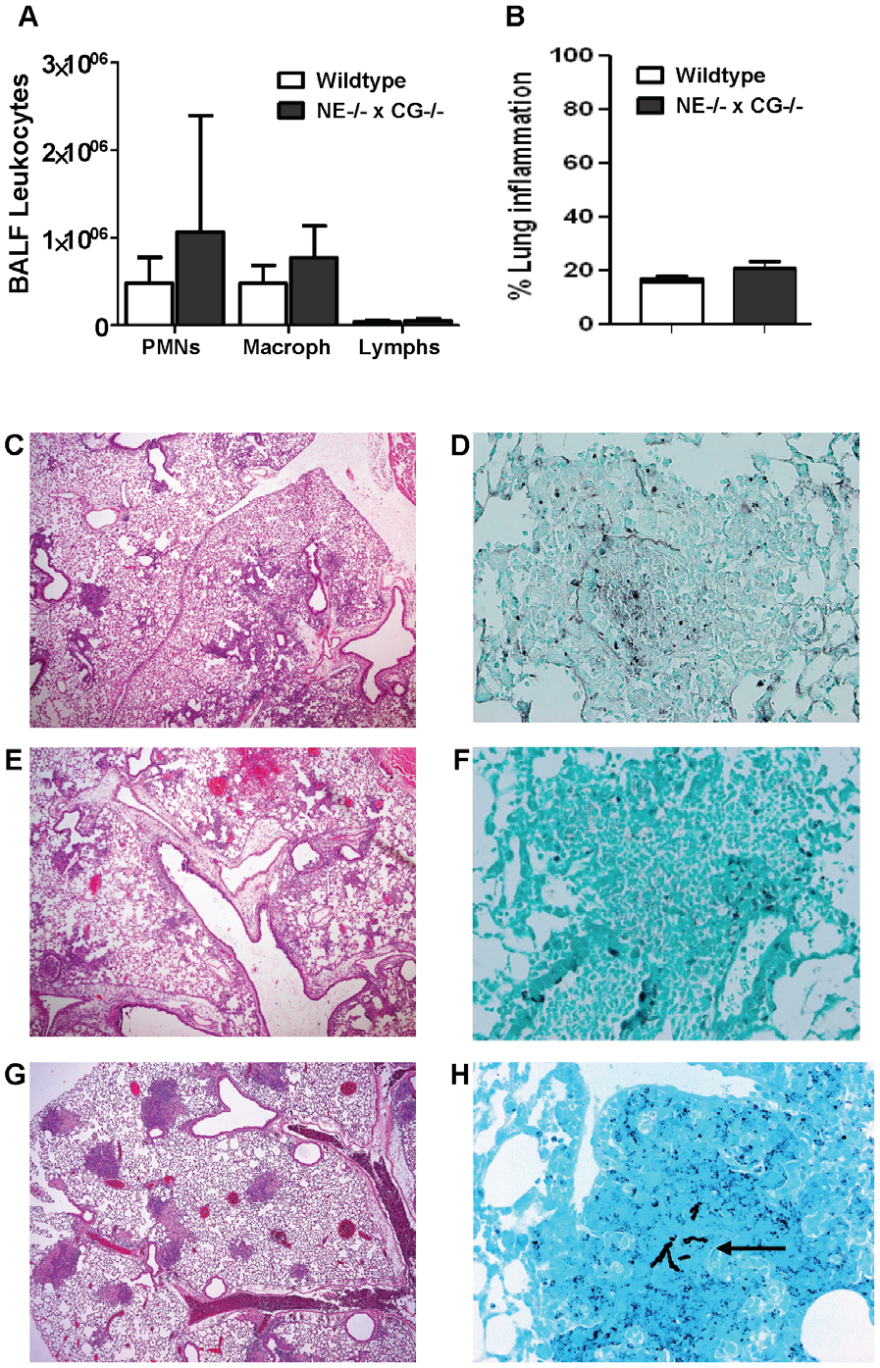
Lung histology and airway inflammation in WT and NE^−/−^×CG^−/−^ mice after *A. fumigatus* administration. Mice were administered *A. fumigatus* (1.25×10^7^ conidia per mouse) by oropharyngeal aspiration and sacrificed on day 3. A) BALF leukocyte recovery and B) percent lung inflammation were similar in WT and NE^−/−^×CG^−/−^ mice. Representative lung histology from WT (C and D) and NE^−/−^×CG^−/−^ mice (E and F). Predominantly peribronchovascular neutrophilic and lymphohistiocytic inflammation occurred in both genotypes (C and E; H&E, 40×). GMS staining (400×) of lung sections from WT (D) and NE^−/−^×CG^−/−^ (F) mice showed what appeared to be degenerated hyphal fragments, but no evidence of intact invasive hyphae. Results are representative of 15 WT and 10 NE^−/−^×CG^−/−^ mice. By comparison, p47*^phox−/−^* mice administered *A. fumigatus* at 0.1% of this inoculum (1.25×10^4^ conidia per mouse) and sacrificed on day 3 had evidence of fungal pneumonia characterized by G) multiple foci of neutrophilic consolidation (H&E, 40×), and H) hyphal parenchymal invasion (arrow) (GMS, 400×).

Since we established that NE and CG deficiency does not recapitulate the CGD phenotype in experimental pulmonary aspergillosis, our subsequent experiments focused on evaluating host defense responses in NE^−/−^×CG^−/−^ mice compared to WT mice. WT and NE^−/−^×CG^−/−^ mice administered a high *A. fumigatus* inoculum (1.25×10^7^ conidia/mouse) were sacrificed on day 3. BALF leukocytosis and extent of lung inflammation were similar between the two genotypes (Figure 2A and B). Lung histology showed a consistent pattern in both genotypes, characterized predominantly by peribronchovascular inflammation (Figure 2 C–F). The inflammatory cell type was mixed, consisting of neutrophils, macrophages and lymphocytes. With GMS staining, we could not identify invasive parenchymal hyphae in any of the lung sections. However, there appeared to be hyphal fragments and debris within inflammatory lesions. Taken together, we could not identify a clear difference in airway or parenchymal inflammation between the genotypes, and, by histological criteria, both genotypes were able to prevent invasive fungal disease. In contrast, p47*^phox−/−^* mice administered a low inoculum (1.25×10^4^ conidia/mouse) developed neutrophilic consolidative lesions associated with invasive fungal disease (Figure 2G and H).

**Figure 3:**
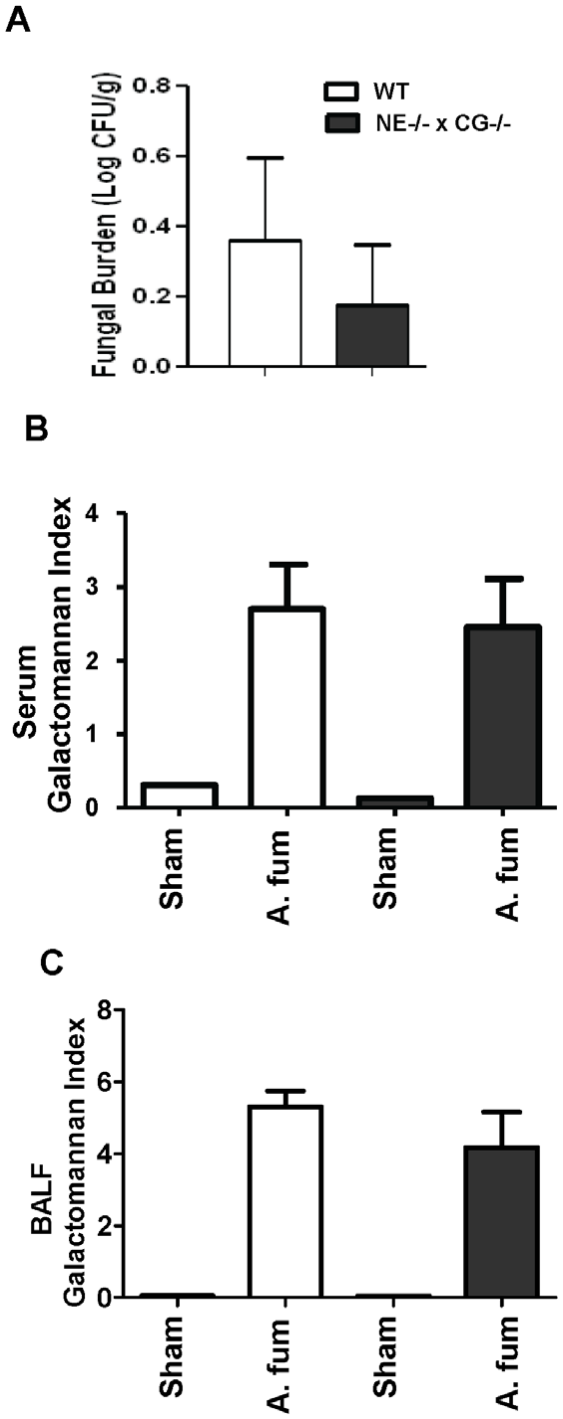
Fungal burden in WT and NE^−/−^×CG^−/−^ mice after *A. fumigatus* administration. Mice were administered *A. fumigatus* (1.25×10^7^ conidia per mouse) by oropharyngeal aspiration and sacrificed on day 3. A) Quantitative fungal cultures in lung homogenates, B) serum galactomannan, C) BALF galactomannan. n = 10 mice per genotype subjected to infection (A. fum), and n = 1 mouse per genotype subjected to sham-infection. No significant differences occurred in quantitative lung fungal cultures, serum galactomannan, and BALF galactomannan between *Aspergillus*-infected WT mice and NE^−/−^×CG^−/−^ mice.

We next performed a more detailed assessment of fungal burden, comparing WT and NE^−/−^×CG^−/−^ mice on day 3 following *A. fumigatus* (1.25×10^7^ conidia/mouse) challenge. There was no significant difference in quantitative fungal cultures of lungs nor was there a difference in serum or BALF galactomannan levels between the genotypes (Figure 3 A–C). Thus, our data point to a dispensable role of NE and CG in host defense against pulmonary aspergillosis.

**Figure 4:**
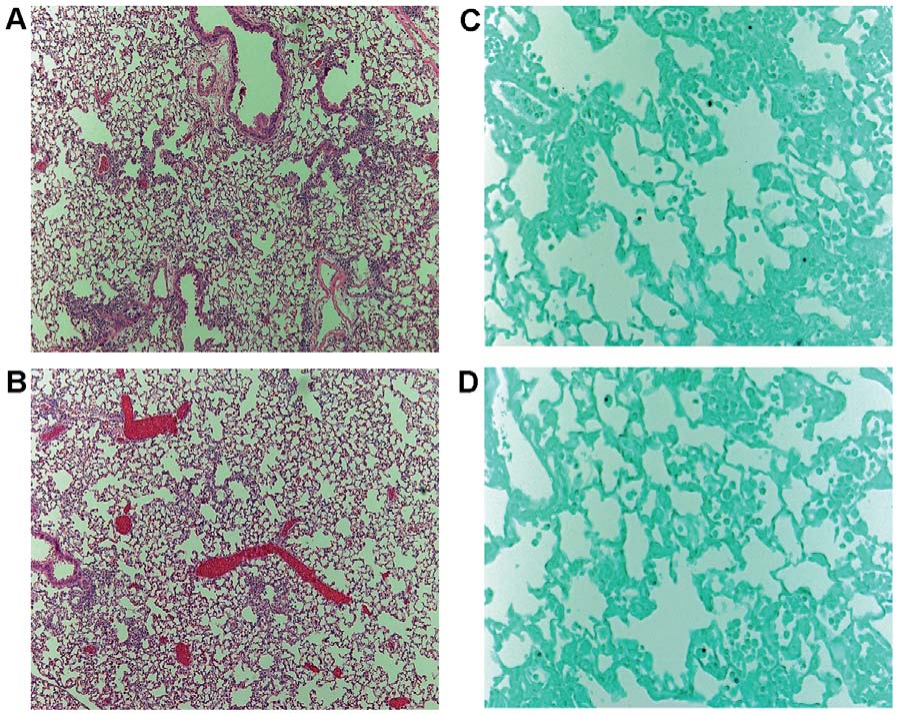
Lung histology in WT and DPPI^−/−^ mice on day 3 after oropharyngeal *A. fumigatus* (1.25×10^7^ conidia per mouse) administration. In both WT (A) and DPPI^−/−^ (B) mice, mild predominantly peribronchovascular inflammation occurred (H&E, 100×). No evidence of invasive hyphae was present with GMS staining (400×) in either WT (C) or DPPI^−/−^ (D) mice. n = 5 mice per genotype.

We considered the possibility that other neutrophil serine proteases may compensate for the lack of NE and CG levels. DPPI is required for the full activation of neutrophil serine proteases, NE, CG, and proteinase 3. Neutrophils from DPPI^−/−^ mice have severe deficiency of these proteases [30]. We administered *A. fumigatus* (1.25×10^7^ conidia/mouse) to WT and DPPI^−/−^ mice and assessed lung histology on day 3. Lung inflammation was mild in both genotypes, predominantly confined to peribronchovascular areas (Figure 4A and B). Similar to NE^−/−^×CG^−/−^ mice, we did not find evidence of hyphal parenchymal invasion in DPPI^−/−^ mice (Figure 4C and D). Thus, using two different neutrophil protease-deficient models, we did not identify a role for these proteases in host defense against pulmonary aspergillosis.

### NADPH is required, but neutrophil elastase and cathepsin G are dispensable, in defense against systemic *Burkholderia cepacia*

We next considered whether NE and CG are required for host defense against systemic *B. cepacia* challenge. *B. cepacia* was selected specifically because it is an important bacterial pathogen in patients with CGD [22], [23] (and patients with cystic fibrosis), but generally not in other immunocompromised patients such as those with prolonged neutropenia or receiving immunosuppressive therapy. Studies of human neutrophils show that killing of *B. cepacia* is NADPH oxidase-dependent [31], and CGD mice have increased susceptibility to *B. cepacia* challenge [28], [32], [33], [34]. Thus, *B. cepacia* infection is an excellent model to evaluate NADPH oxidase-dependent antibacterial host defense.

**Figure 5:**
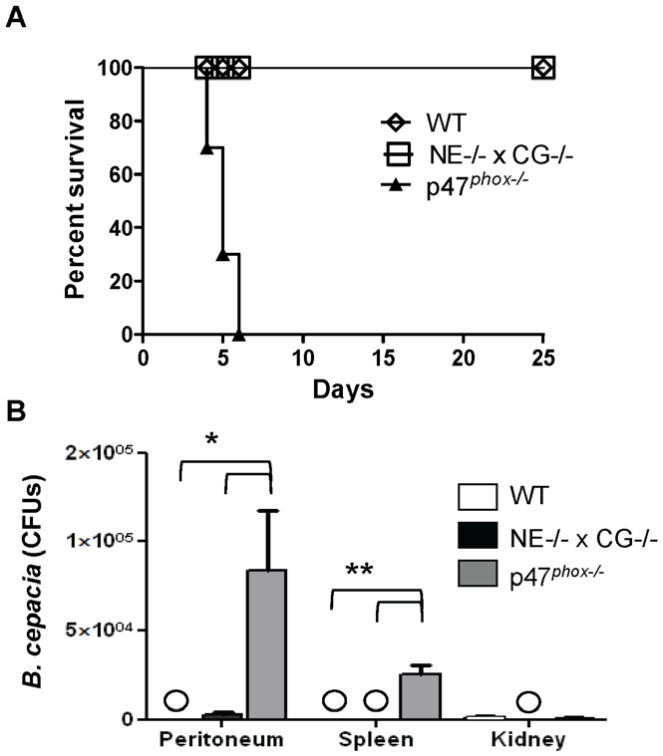
WT mice and NE^−/−^×CG^−/−^ mice were resistant to *Burkholderia cepacia* infection, whereas p47*^phox−/−^* mice were highly susceptible. A) Kaplan-Meier survival curves in WT, p47*^phox^* ^−/−^ and NE^−/−^×CG^−/−^ mice administered intraperitoneal *B. cepacia* (4×10^7^ CFUs/mouse). Log-rank analysis, p<0.0002 comparing WT with p47*^phox^* ^−/−^ mice and p<0.0002 comparing NE^−/−^×CG^−/−^ mice with p47*^phox^* ^−/−^ mice. n = 10 mice per genotype. B) In separate experiments, mice (n = 5 per genotype) were administered the same inoculum of *B. cepacia*, and quantitative cultures were performed at 24 h. WT and NE^−/−^×CG^−/−^ mice cleared infection, whereas bacterial infection persisted in the peritoneum and spleens of p47*^phox^* ^−/−^ mice. Circles, no growth. *, p<0.03; **, p<0.01.

WT, p47*^phox^* ^−/−^ and NE^−/−^×CG^−/−^ mice were administered intraperitoneal 4×10^7^ colony forming units (CFUs) of *B. cepacia*, and time to mortality was followed. Mortality occurred within 4 to 6 days following challenge in p47*^phox^* ^−/−^ mice, whereas all WT and NE^−/−^×CG^−/−^ mice survived (Figure 5A). In separate experiments, mice were administered *B. cepacia* (4×10^7^ CFU), sacrificed at 24 h, and quantitative cultures were performed on blood, peritoneum, kidneys and spleens. Blood cultures from the three genotypes showed no growth. Bacterial recovery from peritoneal cavities and spleens was significantly greater in p47*^phox^* ^−/−^ mice compared to WT and NE^−/−^×CG^−/−^ mice (Figure 5B). Thus, similar to *A. fumigatus*, NADPH oxidase was critical in host defense against *B. cepacia*, while NE and CG were dispensable.

## Discussion

Our results show that NADPH oxidase is essential in defending against *A. fumigatus* and *B. cepacia*, but does so independently of neutrophil serine proteases. In two models of engineered protease-deficient mice, host defense was intact against *A. fumigatus* and *B. cepacia*, two major pathogens in CGD patients. These results do not exclude the possibility that neutrophil serine proteases may have a more central role in host defense against other pathogens that cannot be compensated by NADPH oxidase-dependent pathways. Our results support a model in which NADPH oxidase and neutrophil serine proteases have distinct antibacterial and antifungal effector functions rather than protease activation being the central mechanism by which NADPH oxidase mediates host defense.

Consistent with this notion, patients with CGD and Papillon-Lefèvre syndrome (who lack functional DPPI) suffer from recurrent infections, but the spectrum of pathogens differs between the two diseases. An important consideration is that defects in neutrophil-mediated killing in *ex vivo* studies may not be recapitulated *in vivo* regarding susceptibility to infection. In addition, neutrophil serine protease deficiency may be compensated for by other neutrophil effector pathways. Bianchi et al. [35] identified calprotectin as a NET constituent mediating anti-*Aspergillus* host defense. Indeed, neutrophils are armed with several antimicrobial agents, including lactoferrin [36], lysozyme, and defensins. Potentially, NADPH oxidase-induced serine protease activation may be important for defense against certain pathogens but play a redundant role in defense against others.

There may also be pathogen-related differences that influence susceptibility to neutrophil proteases. Belaaouaj et al. [11] showed an important function of neutrophil elastase in defending against certain Gram-negative (*Klebsiella pneumoniae* and *Escherichia coli*) but not Gram-positive (*Staphylococcus aureus*) bacteria in mice. However, neutrophils from patients with Papillon-Lefèvre syndrome (who lack functional DPPI) do not have a uniform defect in killing *Staphylococcus aureus* and *Escherichia coli*, suggesting that neutrophil serine proteases may not be the major pathway used by human neutrophils to kill specific bacteria [16]. In addition, our experiments focused on acute infection models with early time points for analysis. Potentially, we may have identified differences in host defense between wildtype and protease-deficient mice had we included higher inocula of pathogens and/or different time points for analysis.

Tkalcevic et al. [15] previously showed that NE^−/−^×CG^−/−^ mice were more susceptible to intravenous *A. fumigatus* administration compared to WT mice based on survival and fungal burden in kidneys. This apparent discrepancy with our findings is likely in part related to the route of *Aspergillus* administration. We used oropharyngeal aspiration because inhalation is by far the most common portal of entry of *Aspergillus* species. There are likely critical features of pulmonary host defense that are not reflected in the intravenous model. For example, inhaled conidia are phagocytosed by alveolar macrophages, where NADPH oxidase can play a role in restricting germination of *A. fumigatus* conidia [37]. Neutrophil elastase and cathepsin G can promote coagulation and intravascular thrombus growth *in vivo* that restricts tissue bacterial invasion [38]; conceivably, this pro-thrombogenic effect of neutrophil proteases may limit tissue invasion of intravenously administered fungus, but be less relevant following intrapulmonary challenge. Another difference is the strains of mice used for the studies: we used NE^−/−^×CG^−/−^ mice backcrossed to C57BL/6 whereas the mice used by Tkalcevic et al. [15] were backcrossed to the 129Sv strain. There can be important mouse strain-specific differences in susceptibility to aspergillosis [39] that can influence the relative importance of specific host defense pathways.

Neutrophil serine proteases have also been shown to contribute to pathogen killing through formation of neutrophil extracellular traps (NETs). Upon activation, neutrophils release granule proteins and chromatin that co-mingle in the extracellular environment to form NETs. These NETs bind to and kill bacteria and degrade bacterial virulence factors [40], and target fungi [41], [42]. Release of NETs requires death of neutrophils and breakdown of cell membranes, and has been linked to NADPH oxidase activation and autophagy [43], [44]. NADPH oxidase-mediated NET formation involves complex intracellular signaling, including activation of the Raf-MEK-ERK and upregulation of antiapoptotic proteins [45], and production of interferon-gamma [46]. NET formation was dependent on neutrophil elastase and myeloperoxidase in a mouse model of bacterial pneumonia [47]. Although neutrophils from CGD patients are deficient in NET formation [44], the dependence on NADPH oxidase for NET generation appears to be stimulus-dependent rather than a uniform requirement [48], [49]. Further studies are required to delineate the precise role of NADPH oxidase in NET generation and the role of NETs in NADPH oxidase-dependent pathogen killing.

Histological lung inflammation was similar between WT and neutrophil protease-deficient mice following *A. fumigatus* challenge, a finding that suggests that neutrophil serine proteases do not play a major role in regulating the inflammatory response in this infection model. Prior studies have shown that activation of neutrophil proteases generally leads to augmented inflammation and tissue injury [30], [50], [51], [52], [53]. Although NADPH oxidase activation leads to generation of cytotoxic ROIs, paradoxically, NADPH oxidase limits inflammation and injury in several models [54], [55], [56], [57], [58], [59]. This protective role of NADPH oxidase is likely mediated by modulation of redox-sensitive targets that regulate inflammation and cytoprotective pathways, such as Nrf2 [55]. These results further demonstrate the distinct roles of NADPH oxidase and proteases in modulating inflammation and injury.

NADPH oxidase is a critical regulator of antibacterial and antifungal host defense and of inflammation. Studies of protease-deficient mice and patients with Papillon-Lefèvre syndrome point to neutrophil proteases also having an important host defense function. These results and those of our study support a model in which NADPH oxidase-regulated antimicrobial pathways have distinct pathogen-specific functions in which certain pathogens are sensitive to the direct antimicrobial effect of ROIs whereas others may be controlled by neutrophil serine proteases and other NET constituents.

## Methods

### Ethics statement

All procedures performed on animals in this study were approved by the Animal Care and Use Committee at Roswell Park Cancer Institute, and complied with all state, federal, and NIH regulations.

### Mice

Mice with a targeted disruption of the p47*^phox^* gene have a defective NADPH oxidase, rendering phagocytes incapable of generating measurable superoxide [60]. NADPH oxidase-deficient mice have increased susceptibility to pathogens that afflict CGD patients, including *Aspergillus* species [24], [25], [27] and *B. cepacia* [28], [32], [33], [34]. p47*^phox−/−^* mice were derived from C57BL/6 and 129 intercrosses, and backcrossed 14 generations (N14) in the C57BL/6 background. NE^−/−^ mice [11] and CG^−/−^ mice [14] were intercrossed to generate double knockout NE^−/−^×CG^−/−^ mice (N10 in C57BL/6) [51]. Dipeptidyl peptidase I (DPPI) is a lysosomal cysteine protease required for the activation of granule-associated serine proteases, including NE, CG, and proteinase 3. DPPI^−/−^ mice (N11 in C57BL/6) were generated as previously described [30]. Microsatellite typing performed at the Washington University Rheumatic Disease Core Center's Speed Congenics Laboratory showed the NE^−/−^×CG^−/−^ mice and DPPI^−/−^ mice to be 97.7% and 99.2% C57BL/6, respectively. Age (8–15 weeks) and sex matched C57BL/6 WT mice were used as controls. Mice were bred and maintained under specific pathogen free conditions at the animal care facility at Roswell Park Cancer Institute, Buffalo, NY.

### Administration of *A. fumigatus*

A clinical isolate of *A. fumigatus* was used in all experiments [25]. Conidial suspensions were prepared as previously described [25], diluted to desired concentrations, and administered by oropharyngeal aspiration. We found that oropharyngeal aspiration leads to similar degrees of fungal pneumonia and mortality in p47*^phox−/−^* mice compared to intratracheal administration, but avoids surgery. Mice were anesthetized by isofluorane inhalation using an approved chamber. Following anesthesia, mice were suspended by their upper incisors from a suture thread on a 90° incline board. The tongue was gently extended, and a liquid volume (maximum 50 µl) was delivered into the distal part of the oropharynx. With the tongue extended, the animal was unable to swallow, and the liquid volume was aspirated into the lower respiratory tract. Just prior to liquid delivery, the chest was gently compressed and then released just after deposition of liquid into the oropharynx to enhance aspiration of the liquid into the lung. Mice recovered within 5 minutes of the procedure, and were observed until they resumed normal activity.

### Bronchoalveolar fluid collection and cytology

After sacrifice, BALF collection was performed as previously described [55]. The trachea was cannulated with a 22-gauge i.v. catheter. Using a tuberculin syringe, 1000 µL PBS was injected and withdrawn from the lung and again fresh 1000 µL PBS was injected and withdrawn from the lung and both were pooled. Cells were pelleted by centrifugation at 1,500 g for 3 min. Supernatants were aliquoted and stored at −80°C. In the cell pellet, the red blood cells (RBCs) were removed by ACK lysis, and the cells were suspended in 1 ml of PBS. The total number of leukocytes/ml was counted using a hemocytometer. Cells were then cytocentrifuged onto clean glass slides and stained with the Hema 3 stain set (Fisher Scientific, Pittsburgh, PA, USA), and cell differential counts were assessed blinded to genotype.

### Histopathology

After sacrifice and bronchoalveolar lavage, mouse lungs were infused with 10% neutral buffered formalin via the trachea. Paraffin-embedded blocks were prepared and sections were stained with Hematoxylin and Eosin (H&E) to assess inflammation and Grocott-Gomori methenamine-silver stain (GMS) to visualize fungi. Tissues were microscopically examined for pulmonary injury, vascular invasion, and structural changes in *Aspergillus* hyphae. All slides were analyzed by one of us (BHS) using 40× magnification without formal morphometric analysis, and blinded to genotype. The percentage of lung involved by inflammation was scored in each mouse as follows: 0%, 5%, 10%, and then by 10% increments (e.g., 20%, 30%, 40%, etc.). The predominant inflammatory cell type was scored.

### Assessment of fungal burden

Fungal burden was assessed in four ways. (i) The presence of invasive hyphae in lungs was determined by histology. (ii) Quantitative cultures of lung homogenates were performed using a previously validated method [61]. Lungs were weighed, placed in a sterile polyethylene bag (Tekmar Corp., Cincinnati, Ohio), and homogenized with sterile saline for 30 s (Stomacher 80; Tekmar Corp., Cincinnati, Ohio). Lung homogenate dilutions (10^−1^ and 10^−2^) were prepared in sterile saline. Aliquots (100 µl) from homogenates and homogenate dilutions were plated on Sabouraud glucose agar plates, incubated at 37°C for the first 24 h, and then left at room temperature for another 24 h. The number of colony forming units (CFUs) of *A. fumigatus* was counted, and the CFUs per gram was calculated. A finding of one colony of *A. fumigatus* was considered positive. (iii) Galactomannan is a fungal cell wall product. The serum concentration of galactomannan is directly related to the concentration of *A. fumigatus* in lung tissue of experimental invasive pulmonary aspergillosis [62], [63]. Detection of galactomannan is used clinically as a diagnostic adjunct for invasive aspergillosis [64]. Batched frozen serum samples from infected and sham-infected mice were thawed, and galactomannan levels were quantitated using Platelia *Aspergillus* enzyme immunoassay (Bio-Rad Laboratories, Redmond, WA) per the manufacturer's instructions. (iv) BALF galactomannan levels were determined using the same assay. Galactomannan concentrations in BALF also correlate directly with concentrations of *A. fumigatus* in lung tissue [65].

### Administration of *Burkholderia cepacia*

We used a *B. cepacia* strain isolated from a CGD patient [33]. Bacteria were stored in Lennox broth (Invitrogen) at −80°C until use. Aliquots of frozen bacteria were thawed, inoculated into Lennox broth, and grown with shaking at 37°C overnight. The bacteria were washed twice in DPBS, and bacterial density was determined by absorption at 650 nm at dilutions corresponding to the linear portion of the absorbance-bacterial density curve. To confirm the bacterial density, aliquots were serially diluted and sub-cultured in duplicate on Lennox agar plates, and colony forming units were counted. Mice were injected intraperitoneally with 0.2 ml of bacteria at the desired concentration. One set of mice was followed for survival, while bacterial clearance was assessed 24 hours after challenge in a second set of mice. In this second set of mice, blood was collected retro-orbitally into Z (no additive tube) microtainers (BD Biosciences). Peritoneal lavage was performed with 10 ml of DPBS, and spleens and kidneys were harvested and homogenized in 2 ml of DPBS. Samples were diluted in serial 10-fold dilutions and inoculated in duplicate onto Lennox agar plates. After inoculation, plates were incubated for 24 to 48 h at 37°C and colonies were enumerated.

### Time to euthanasia

Following infection, mice were monitored twice daily for death and morbidity until at least day 25. Mice with pre-specified criteria for distress that included inability to feed or drink, labored breathing, or general moribund appearance were euthanized by CO_2_ asphyxiation.

### Statistical Analysis

Kaplan-Meier curves were generated for each population for time to euthanasia experiments (Graph Pad Prism 4.0) and analyzed using the log-rank method. In experiments involving quantitation of bacterial and fungal burden, inter-group comparisons were made using the non-parametric Mann-Whitney method. In cases in which bacterial cultures were sterile in all mice in a given group, Wilcoxon Signed Rank test was used for inter-group comparisons. A two-sided p value of <0.05 was considered to be statistically significant.
